# Role of Gut Microbiota and Metabolite Remodeling on the Development and Management of Rheumatoid Arthritis: A Narrative Review

**DOI:** 10.3390/vetsci12070642

**Published:** 2025-07-05

**Authors:** Yichen Yu, Fulin Jin, Lijun Wang, Ji Cheng, Shifeng Pan

**Affiliations:** 1 College of Veterinary Medicine, Yangzhou University, Yangzhou 225009, China; yuyichen1608@163.com (Y.Y.); 13851118926@163.com (F.J.); wlj6379@163.com (L.W.); 008743@yzu.edu.cn (J.C.); 2 Department of Animal Sciences, Washington State University, Pullman, WA 99163, USA; 3 Guangling College, Yangzhou University, Yangzhou 225009, China; 4 Jiangsu Co-Innovation Center for Prevention and Control of Important Animal Infectious Diseases and Zoonoses, Yangzhou University, Yangzhou 225009, China

**Keywords:** rheumatoid arthritis, intestinal microbiota, animal production, intestinal barrier, immune response, probiotic therapy

## Abstract

Rheumatoid arthritis (RA) is a chronic autoimmune disease that has a serious impact on both human health and animal production. The gut microbiota is a large and complex symbiotic ecosystem in animals, and the imbalance of gut microbiota is closely related to the pathogenesis of numerous diseases, including RA. The interactions among the gut microbiota, intestinal barrier, and immune system play key roles in maintaining intestinal homeostasis and affecting the development of RA. Regulating intestinal flora and metabolites provides new ideas for the prevention and treatment of RA. Probiotics can regulate the balance of intestinal flora and metabolites, improve the immune environment, and provide novel therapeutic strategies against RA. In order to summarize the role of gut microbiota and metabolite remodeling in the development and management of RA, this review will elaborate on the role of intestinal flora imbalance in the pathogenesis of RA and assess prospective therapeutic approaches that target the gut flora. Understanding the interaction among intestinal flora, metabolites, and RA will help to clarify the pathogenesis of RA and develop innovative and personalized therapeutic interventions against chronic autoimmune diseases.

## Simple Summary

Rheumatoid arthritis is a chronic autoimmune disease that causes joint pain, inflammation, and disability in humans and animals, harming health and livestock productivity. This review explores the growing evidence linking rheumatoid arthritis to imbalances in the gut microbiome—the trillions of bacteria and microbes living in the intestines. The goal is to understand how these microbial changes contribute to rheumatoid arthritis and whether modifying the gut microbiome could offer new treatment strategies. Studies show RA patients and animals have fewer beneficial gut bacteria and more harmful bacteria. These imbalances weaken the gut barrier, allowing harmful substances to enter the bloodstream and spark systemic inflammation. The gut microbiome also disrupts immune balance, promoting damaging inflammation while reducing protective responses. Interventions like probiotics and fecal microbiota transplantation improved gut health and reduced rheumatoid arthritis symptoms in animal studies. By highlighting the gut–rheumatoid arthritis connection, this review suggests targeting gut health could provide affordable, low-risk treatments, especially for animals with limited therapies. The findings may inspire new probiotic products or personalized microbial treatments to prevent rheumatoid arthritis progression, benefiting both human patients and global livestock health.

## 1. Introduction

Rheumatoid arthritis (RA) is a chronic autoimmune disease characterized by joint stiffness, pain, and limited mobility. It can lead to a great loss of joint function and, in severe cases, disability or involvement of other tissues and organs. The occurrence of RA seriously affects both human health and the development of livestock and poultry.

At present, RA research in animals is relatively scarce, which seriously hinders the development of effective treatment strategies against RA in animals and causes a lack of specific therapeutic drugs and methods. Additionally, due to the differences in the immune systems of animals from those of humans [1], the direct application of human drugs to animals may result in many difficulties in determining drug doses and preventing adverse reactions [2]. Furthermore, the treatment of RA in animals faces challenges in terms of economic cost and breeder awareness [3].

An increasing number of studies have demonstrated that multiple therapies based on altering gut microbiota diversity can play a vital role in treating RA, suggesting that gut microbiota remodeling can be a more cost-effective option in RA prevention and treatment [4,5]. At the preventive level, the balance of the intestinal microbial community can be used as a target for early intervention in RA, with the advantages of being relatively inexpensive and easy to implement. In terms of disease relief, the regulatory effect of gut microbial remodeling on metabolism helps to reduce joint inflammation and improve feed conversion and has relatively few side effects and adverse reactions, such as drug residue [6]. In terms of long-term benefits, the plasticity of gut microbes offers the possibility of sustainable RA management. Through continuous microbial community monitoring and targeted interventions, intestinal homeostasis may be maintained and RA recurrence may be prevented.

This article aims to provide a synthesis of recent research findings on the relationship between RA and the gut microbiota and will explore the role of gut microbiota imbalance in the pathogenesis of RA and the potential applications of gut microbiota regulation in the treatment of RA, to better aid the development of novel therapeutic approaches against RA.

## 2. The Compositional and Functional Imbalance of the Gut Microbiota in Patients and Animals with RA

The gut microbiota represents an extremely large and complex symbiotic microbial ecosystem in animals. These microorganisms not only participate in the digestive and absorptive processes of food but also exert a profound impact on the health of the host through their metabolites [7,8]. The gut microbiota produces a wide variety of metabolites, including short-chain fatty acids (SCFAs), amino acids, and so forth. With the advancement of microbiome research, these metabolites have been regarded as a crucial link between gut microbes and host health [9].

In recent years, an increasing number of studies have demonstrated that the gut microbiota and derived metabolites play a pivotal role in the pathogenesis and/or progression of arthritis [10,11]. The compositional and functional imbalance of the gut microbiota has been summarized in animals with inflammatory arthritis (Table 1) and RA patients (Table 2).

## 3. The Role of Intestinal Flora Imbalance in the Pathogenesis of RA

The mutualism between microbiota and their host is a symbiotic relationship beneficial to both parties. An imbalance between intestinal microbiota and the host can potentially lead to the onset of serious diseases that pose a significant threat to human and animal health [20].

### 3.1. Relationship Between RA and Gut Microbiota, Gut Microbiota Metabolites, and Gut Barrier

There is a complex and delicate equilibrium between the gut microbiota and its metabolites and the intestinal barrier. On the one hand, the intestinal flora produces beneficial metabolites through the fermentation of indigestible carbohydrates, including dietary fiber [21,22]. These metabolites can provide energy for intestinal epithelial cells and promote the repair and regeneration of the intestinal mucosa, as well as enhance the integrity of the intestinal barrier [23,24]. Concurrently, metabolites can regulate the activity of intestinal immune cells and inhibit the inflammatory response, thereby maintaining intestinal health [25,26]. On the other hand, gut integrity is of paramount importance in preventing the infiltration of harmful substances into the bloodstream. A disruption to the intestinal barrier function, such as an increase in permeability, will give rise to a systemic inflammatory and immune response in the host, the persistence of which will not only exacerbate the damage to the intestine itself but also affect the function of other organ systems, such as triggering autoimmune diseases [27].

Equilibrium disruptions of the gut microbiota are frequently observed in patients and animals with RA, accompanied by an increase in intestinal permeability and a reduction in the integrity of the intestinal barrier. ZO-1, Cluadin-1, and Occludin are key tight junction proteins and have important effects on the integrity and function of the intestinal barrier. A cross-sectional study demonstrated a correlation between fecal tight junction protein levels in RA patients and RA clinical parameters. Among these parameters, Claudin-1 level was found to be significantly decreased, resulting in altered intestinal permeability in patients [28]. The study by Wen et al. found that at the onset of RA, there was a peak in the expression of intestinal hypoxia-inducible factor (HIF)-2α in intestinal epithelial cells, which in turn induced the transcription of Claudin-15, thereby disrupting the intestinal barrier and promoting the development of arthritis [29]. Zonulin is a peptide hormone secreted by intestinal epithelial cells, whose core function is to regulate the dynamic opening and closing of intestinal tight junctions to maintain selective permeability of the intestinal barrier. Elevated levels of Zonulin are often observed in the serum of individuals at risk for RA and in RA patients, and this alteration is accompanied by leakage of the intestinal barrier, microbial community imbalance, and inflammation [30]. Therefore, maintaining the balance of intestinal flora and the integrity of the intestinal barrier function is of great importance in the prevention and treatment of RA.

### 3.2. Interactions Among RA, Gut Microbiota, and Immune System

The immune system plays a pivotal role in the pathogenesis of RA. Dendritic cells (DCs) present antigens to naive T cells and induce them to differentiate into different cell subtypes (Th1, Th17, and Treg cells, etc.) under the action of antigen-stimulating signal factors and produce related cytokines or immune tolerance. Th1 cells activate macrophages and are responsible for cell-mediated immunity and phagocyte-dependent protective responses. The prevailing theory posits that the manifestation of RA is contingent on the over-expansion and expression of Th1 cells and its hallmark factor, IFN-γ.

The discovery of a new subset of T cells, Th17 cells, has led to the establishment of a link between Th17 cell plasticity and autoimmune diseases [31]. Research has demonstrated that Th17 cells, the sole osteoclast-derived Th cell subset, along with its hallmark factor, IL-17A, assume a pivotal role in bone destruction in RA [32]. The release of inflammatory cytokines (TNF-α, IL-1β, and IL-6), chemokines, and matrix metalloproteinases are promoted by IL-17A, which can also promote the formation and activation of osteoclasts [33]. TNF-α has been demonstrated to up-regulate the expression of secreted proteins in synovial fibroblasts, thereby inhibiting Wnt signaling, which is required for bone formation in osteoblasts [34]. Activation of the TNF-α pathway in CD4+ T cells is critical for the development of RA [35]. These cytokines induce a high responsiveness of osteoclast precursor cells to the receptor activator for nuclear factor-κB ligand (RANKL) and up-regulate RANKL expression in synovial fibroblasts [36]. This process culminates in the amplification of osteoclastogenesis.

In contrast, the function of Treg cells is characterized by their ability to impede RANKL-induced osteoclastogenesis and increase bone volume in vivo [37], thereby exerting a pivotal role in the initiation and management of RA [38,39]. This function is facilitated by the involvement of TGF-β/IL-4, TGF-β/IL-10, or CTLA-4, operating through either a contact-dependent or -independent cell-to-cell interaction. CTLA-4 has been shown to bind to the osteoclast precursor CD80/CD86, inducing osteoclast apoptosis. These findings suggest that the balance between Treg cells and Th17 cells may be a key factor in the inhibition of bone destruction in RA.

It is noteworthy that an imbalance in the gut microbiota and the production of harmful metabolites may be a significant contributing factor to the aberrant immune system response. Fecal microbiota transplantation (FMT) from patients at high risk of developing RA results in an intestinal muco-immune imbalance in mice, characterized by an increase in Th17 cells and elevated mRNA levels of IL-17a, IL-22, and TNF-α in the small intestine [40]. Maeda et al. observed that mice with the microbiota of RA patients exhibited elevated numbers of intestinal Th17 cells and developed severe arthritis following zymosan injections. Furthermore, these mice exhibited augmented IL-17 responses in lymphocytes [41]. The study conducted by Jubair et al. observed marked dysbiosis and mucosal inflammation at an early stage of collagen-induced arthritis (CIA). In addition, depletion of the microbiota was observed to have a mitigating effect on both the severity of inflammation and the progression of disease in both the early and late stages of CIA. This provides further confirmation of the significant role played by the gut microbiota in the pathogenesis of RA [42].

### 3.3. Relationship Between Specific Bacterial Species and RA

A substantial body of research has demonstrated that particular bacterial species, including *Escherichia coli*, are linked to the onset of RA. It has been demonstrated that the elevation of *E. coli* enhances the arginine succinyl transferase pathway in RA stages II and III, which is associated with the elevation of the rheumatoid factor and can induce bone loss [19]. It is possible that this effect of *E. coli* is closely related to the production of lipopolysaccharide (LPS). As early as 1994, it was demonstrated that LPS produced by *E. coli* induces arthritis in rats at a similar rate to that of *E. coli* itself. Furthermore, local stimulation of IL-1 production may play an important role in this process [43]. In the study conducted by Kitamura et al., the administration of LPS produced by *E. coli* resulted in the destruction of articular cartilage and the infiltration of inflammatory cells in mice, leading to the development of severe arthritis [44]. Consequently, *E. coli* or its LPS is frequently employed in the construction of RA models both in vitro and in vivo [45].

In contrast to LPS from *E. coli*, LPS from *Bacteroides fragilis* demonstrated a diminished capacity to induce arthritis. Furthermore, it exhibited the ability to impede the inflammatory response initiated by LPS from *E. coli,* thereby inhibiting the progression of arthritis [44]. It is hypothesized that this effect may be achieved by promoting the development of intestinal Treg cells and inhibiting the immune responses of intestinal Th17 cells [46]. Furthermore, the cell wall lipoprotein of *Bifidobacterium longum* has been demonstrated to exert a significant influence on the prevention of arthritis via the gut–joint axis [47]. *Lactobacillus rhamnosus* has been demonstrated to alleviate RA symptoms by reducing inflammation mediated by CD8+ T cells [48].

### 3.4. Molecular Simulation Is a Potential Mechanism for Connecting RA with Intestinal Flora

Molecular mimicry refers to the phenomenon whereby antigens produced by specific pathogens or gut microbes that share sequence homology with autoantigens may lead to cross-activation of autoreactive T or B cells, thereby triggering an autoimmune response [49]. At present, the roles of several molecular mimicry mechanisms related to the intestinal microbiota in the occurrence of RA have been revealed.

Protein citrullination is a post-translational modification process that requires peptidylarginine deiminase (PAD). While this is a normal physiological process and not unique to RA, citrullination of RA autoantigen proteins by PAD is a key event in the pathogenesis of RA [50]. Furthermore, PADI4 is also found to be highly expressed in the synovial membrane of RA joints [51]. A variety of anti-citrullinated protein antibodies can be detected in the serum of patients prior to the onset of RA, and these antibodies form immune complexes with corresponding antigens in the joints and deposit in the synovium, causing synovial inflammation. It has been hypothesized that in individuals with a predisposing genetic background, citrullinated protein antigens disrupt immune tolerance, thereby contributing to the pathogenesis of RA [52]. The structure of cholesterol-dependent cytolysin secreted by some Gram-positive bacteria is similar to that of perforin-membrane attack complex family proteins, which can promote Ca^2+^ influx through the formation of perforin to activate endogenous PAD enzymes and participate in citrullination [50].

A significant number of studies have recently concentrated on the role of autoantigen citrullination mediated by *Porphyromonas gingivalis*. Unlike other PAD, which requires Ca^2+^ to catalyze the conversion of peptidyl arginine to citrulline [53], PAD secreted by *P. gingivalis* can catalyze the citrullination in the absence of Ca^2+^, resulting in the production of RA-specific autoantigens [54]. These findings suggest that *P. gingivalis* may break the host immune tolerance to citrullinated proteins and induce local immune responses in the joints.

Neutrophils represent the most prevalent cell type in RA synovial fluid and have the capacity to infiltrate synovial tissue. Certain bacterial species have been observed to induce the formation of neutrophil extracellular traps (NETs) [55], a process that involves the release of the PAD enzyme to trigger PAD activation and modify the histone arginine residues in the neutrophil nucleus to citrulline [56,57]. Studies have revealed that RA patients exhibit increased levels of citrullinated histone H3 in their neutrophil populations. Antibodies present in the serum of RA patients have been observed to bind to citrullinated histone H4, a by-product of NETs [58]. Furthermore, it has been determined that the activation of NADPH oxidase (NOX) is a prerequisite for the formation of NETs [59]. It has been established that the Gram-positive bacteria *Lactobacillus* spp. can stimulate the activation of NOX1 [60], while certain bacteria such as *E. coli* have been observed to activate AMPK and inhibit NOX activation [61,62]. Consequently, these bacteria may possess the capacity to affect NET formation.

In RA patients and animals, disruption of the gut microbiota may result in aberrant expression of specific microbial antigens that cross-react with RA-related autoantigens through molecular mimicry mechanisms. Specific phages that are enriched in the gut of RA patients and animals, particularly those derived from phages *Prevotella* and *Oscillibacter*, were found to have a high degree of homology with the epitopes of the RA autoantigen BiP. These phage peptides have the capacity to activate CD4+ T cells and B cells through the mechanism of molecular mimicry, thereby triggering an autoimmune response and promoting the occurrence and development of RA [63]. Additionally, the disruption of the intestinal phagome in RA patients is significantly associated with the presence of disease-specific autoantibodies and disease-susceptibility genes (such as HLA-DR shared epitopes). The phage composition of RA patients with an HLA-DR susceptibility gene differs from that of patients without, indicating a potential correlation between intestinal phage and disease susceptibility genes in RA [63]. These findings offer new insights into the pathogenesis of RA and may provide new avenues for immunotherapy in the future.

## 4. Application of Intestinal Flora Regulation in the Treatment of RA

In recent years, there have been encouraging indications that oral probiotics and FMT may prove to be effective as adjunctive treatments for RA.

### 4.1. Therapeutic Effects of Probiotics on RA

**Figure 1:**
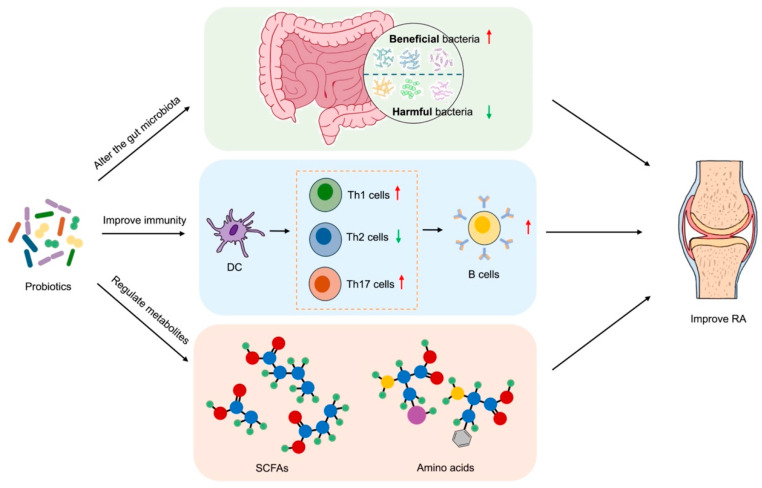
Therapeutic effects of probiotics on RA. Probiotics modulate the gut microbiota by promoting the proliferation of beneficial bacteria and inhibiting harmful bacteria, thereby restoring intestinal flora balance. This modulation regulates immune responses by reducing the differentiation of pro-inflammatory Th1/Th17 cells and enhancing anti-inflammatory Th2 cells and Tregs. Concurrently, probiotics ferment dietary fiber to produce SCFAs and other metabolites, which improve intestinal barrier integrity and suppress systemic inflammation. The combined effects of microbiota–immune–metabolite interactions ultimately alleviate RA symptoms. Red arrows represent promotive effects, and green arrows represent inhibitory effects. PowerPoint was used to draw this figure.

Probiotics, as modulators of gut microbiota, have demonstrated efficacy in the treatment of RA (Figure 1).

Probiotics have the capacity to regulate the equilibrium of intestinal flora by augmenting the number of beneficial bacteria and impeding the proliferation of harmful bacteria. In the study by Pan et al., treatment with *Lactobacillus casei* increased the abundance of *Lactobacillus acidophilus*, *Lactobacillus hominis,* and *Lactobacillus vaginalis* and then improved arthritis [64]. In addition to competing for nutrients and living space, probiotics are known to produce antibacterial substances, which serve to inhibit the growth and reproduction of harmful bacteria. It has been demonstrated that probiotics entering the gut will automatically aggregate under the mediation of extracellular polysaccharides and hydrophobic surface proteins. They will then adhere to epithelial cells with a stronger adhesion force through competitive repulsion, thereby reducing the adhesion of pathogenic bacteria [65]. For example, *Bacillus coagulans* may eliminate the antagonistic microorganisms that cause inflammatory responses by producing bacteriocins and L(+) lactic and competing for the adhesion sites of the mucosa, thereby exerting a protective effect on RA patients [66].

Probiotics have been demonstrated to stimulate the activation and proliferation of immune cells, including macrophages, DCs, and T lymphocytes, as well as regulate the production of cytokines within the intestine. Reduced production of pro-inflammatory cytokines, such as TNF-α, IL-1β, and IL-6, and increased secretion of anti-inflammatory cytokines, including IL-10 and IL-4, can help to reduce the inflammatory response in the gut and the level of systemic inflammatory response, exerting a beneficial influence on the treatment of RA. Qin et al. found that *Lactiplantibacillus plantarum* was effective in improving autoantibody levels, cytokine imbalance, and oxidative stress in RA mice [67]. *Saccharomyces boulardii* has been demonstrated to reduce the levels of pro-inflammatory cytokines, inhibit the proliferation of Th17 and type 3 innate lymphocytes (ILC3), and regulate immune disorder in arthritis rats [68]. Furthermore, *L. rhamnosus* GR-1 and *Lactobacillus* reuteri RC-14 reduce the serum levels of IL-1α, IL-6, IL-12p70, and TNF-α in RA patients and have a positive anti-inflammatory effect at the cellular level [69]. Similarly, *L. casei* treatment can also regulate the production of cytokines and reduce lymphocyte infiltration at the same time, thereby inhibiting the development of arthritis [70].

Probiotics have also been demonstrated to exert a beneficial impact on the management of RA by modulating the production of metabolites within the gut microbiota (Table 3).

Probiotics are capable of fermenting dietary fiber to produce SCFAs, including acetic acid, propionic acid, and butyric acid. This process reduces the pH value in the gut and inhibits the growth and reproduction of harmful bacteria. Concurrently, SCFAs can facilitate the restoration of the intestinal mucosal barrier and augment the defensive capabilities of the intestinal mucosal barrier. SCFAs play an immunomodulatory role mainly by promoting the differentiation of Treg cells; in particular, butyric acid regulates the balance of Treg/Th17 cells [81,82,83], so it also has a protective effect on joint inflammation and damage. A reduction in butyric acid-producing bacteria, such as *Faecalibacterium*, was observed in RA patients. The exogenous intake of butyric acid-producing probiotics has been demonstrated to supplement butyrate, promote the differentiation of Treg cells, inhibit that of Th17 cells and T follicular helper cells, and regulate the Th17/Treg balance, which in turn mitigates the severity of arthritis [84].

It is estimated that approximately 95% of human serotonin (5-hydroxytryptamine, 5-HT) is produced in the gastrointestinal tract. Rodent and human innate immune cells have been shown to express 5-HT receptors, and intra-articular injection of 5-HT in mice has been observed to cause joint inflammation and pain, whereas depletion of 5-HT has been demonstrated to reduce disease severity [85]. Research has demonstrated that the ingestion of probiotics exerts an influence on 5-HT production and function. *B. longum* subsp. *infantis* B6MNI modulates the gut microbiota and fecal metabolites, including 5-hydroxyindole-3-acetic acid (5-HIAA), to affect Pim-1 expression and immune cell differentiation. This then affects joint inflammation, regulates osteoclast differentiation factors, and delays the progression of RA through the JAK/STAT3 pathway [86].

It is worth noting that the safety of using probiotics remains a potential issue. Theoretically, there could be four kinds of side effects: systemic infections, deleterious metabolic activities, excessive immune stimulation in susceptible individuals, and gene transfer [87]. Therefore, more research is needed to confirm these points.

### 4.2. Intestinal Flora Transplantation and RA Treatment

FMT is a therapeutic modality that involves the transfer of the intestinal flora of healthy individuals into the intestinal tract of patients. FMT has demonstrated considerable efficacy in the treatment of intestinal disorders, such as *Clostridium difficile* infection [88], and is increasingly being employed in the management of other conditions, including RA. In 2020, Zeng and colleagues reported the first successful case of FMT for RA [89].

Although there are still many issues to be explored and solved in practical application, such as the possible occurrence of adverse reactions like abdominal distension, diarrhea and constipation [90,91], the prospect of FMT in treating RA remains optimistic. For example, the efficacy and safety of FMT can be further improved by optimizing the donor screening criteria and improving the accuracy [92]. Moreover, FMT can be used in combination with other treatment modalities, such as immunosuppressants and non-steroidal anti-inflammatory drugs [93], and this comprehensive treatment plan is expected to further improve the treatment effect of RA. In addition to treating RA, FMT treatment in high-risk groups may restore the balance of intestinal flora and normal immune function, thereby reducing the incidence of RA.

### 4.3. Interaction Between Intestinal Flora and RA Therapeutics

**Figure 2:**
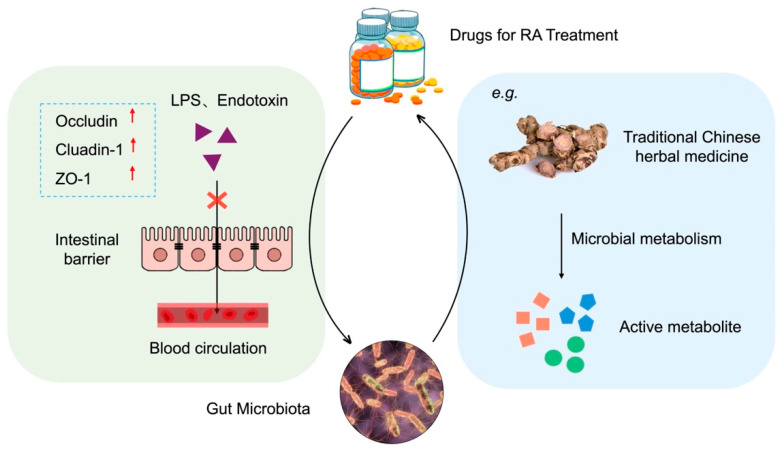
Interactions between gut microbiota and therapeutic drugs for RA. **Left**: Drugs regulate gut microbiota composition and enhance intestinal barrier function by upregulating tight junction proteins (Occludin, ZO-1, Claudin-1). **Right**: Gut microbiota metabolize drugs into active compounds or influence drug bioavailability. The bidirectional arrow indicates dynamic interactions between drugs and microbiota. PowerPoint was used to draw this figure.

There are numerous interactions between the gut microbiota and various drugs used in the treatment of RA (Figure 2).

On the one hand, pharmaceutical agents exert regulatory effects on the gut microbiota. Studies have demonstrated that certain drugs can exert a therapeutic effect by enhancing the composition of the intestinal flora and reinforcing the intestinal mucosal barrier. Jingfang Granules have been observed to augment the prevalence of intestinal microbiota, consequently elevating the concentration of SCFAs in the intestine and serum of RA rats, which has been shown to activate AMPK, regulate fatty acid metabolism and biosynthesis, and thereby mitigate RA-induced tissue damage [94]. Methotrexate (MTX) is currently the primary synthetic drug used in the treatment of RA. It has been demonstrated that MTX alters the human gut microbiota, resulting in reduced host immune activation and consequently providing protection against RA [95].

On the other hand, the gut microbiota exerts a significant influence on the metabolism of drugs. Some pharmaceutical ingredients, particularly traditional Chinese herbs, are metabolized by intestinal flora into active compounds in the gut, thereby exerting therapeutic effects. For example, O-desmethylangolensin, 3-hydroxydodecanedioic acid, and other metabolites produced by the fermentation of *Lycium barbarum* polysaccharide by gut flora may be the key to alleviating RA [96]. Similarly, *L. casei* 3260-fermented *Gleditsia sinensis* thorn extract can significantly reduce cartilage damage caused by RA [97]. Furthermore, the gut microbiota affects the metabolism and excretion of drugs, which in turn impacts the bioavailability and efficacy of drugs and, ultimately, their therapeutic effect. Zhou et al. observed that the absence of *B. fragilis* resulted in the absence of a significant therapeutic effect of MTX. Conversely, the transplantation of *B. fragilis* restored the efficacy of MTX [98].

It is worth noting that diet, as a non-pharmaceutical intervention method, is an effective approach for the long-term management of diseases. The role of anti-inflammatory diets characterized by low calorie levels and rich in omega-3 fatty acids in the prevention and treatment of RA is receiving increasing attention. Studies conducted among obese and non-obese subjects have shown that calorie restriction can reduce the level of TNF—α in serum [99], as well as the expression of NLRP3 inflammasome and il-1b in adipose tissue [100]. Omega-3 polyunsaturated fatty acids in the diet can directly regulate the types and abundance of intestinal microbiota, change the levels of pro-inflammatory mediators, and regulate the levels of microbiota metabolites. For example, it can reduce the growth of Enterobacteria, increase the growth of *Bifidobacteria*, *Blautia*, etc., and subsequently inhibit the inflammatory response related to endotoxemia and increase the content of SCFA [101,102]. Correspondingly, certain microorganisms, such as *Bifidobacterium*, can also promote the absorption of Omega-3 polyunsaturated fatty acids [103]. This interaction may have a positive effect on reducing inflammation and RA management.

### 4.4. Summary of Therapeutic Interventions Targeting Gut Microbiota in RA Management

Table 4 summarizes the effects of the intervention measures mentioned herein for RA. These findings highlight the translational potential of microbiota-based therapies for veterinary applications.

### 4.5. Safety Considerations and Practical Suggestions for Microbial Therapy in Veterinary RA Treatment

Microbial intervention measures have shown promising therapeutic potential in the treatment of animal RA by regulating the intestinal microbiota and the immune system. However, the potential safety risks associated with them still warrant special attention.

Intestinal barrier damage often exacerbates the inflammatory cascade through complex mechanisms, which are of great significance for the safety of microbial therapies in veterinary RA treatment. Species-specific factors are crucial, as the structure and immune response of the intestinal barrier vary among different species, which affects the risk of systemic inflammation. For example, the higher immune sensitivity of dogs makes them more susceptible to inflammation as caused by barrier damage [105]. Similarly, the fragile intestinal barrier of poultry and the complex rumen ecosystem of ruminants require targeted microbial intervention measures to minimize the risks of bacterial dysbiosis and barrier dysfunction. Therefore, although microbial therapies have the potential to regulate immune responses and reduce inflammation, precise control is necessary to avoid unintended immune activation [106,107].

In the treatment of RA, microbial therapies present a paradoxical phenomenon in treatment: they can both alleviate and exacerbate immune imbalance, potentially leading to symptom deterioration. In particular, probiotic therapy may overstimulate the immune system, especially in individuals with existing immune imbalance, resulting in the production of autoantibodies and the activation of autoreactive T cells, further exacerbating the autoimmune response and worsening RA symptoms. This therapeutic contradiction factor becomes even more complex due to the specific responses of different species to microbial intervention [108,109]. Some microbial strains may enhance regulatory immunity in one species but trigger adverse immune responses in another species. Current methods cannot precisely regulate microbial activity, which further exacerbates this challenge and limits the precision of microbial therapies.

Furthermore, there are serious safety issues with the interaction between microbial therapies and conventional drugs, as there may be cross-toxicity and adverse reactions. Microbial intervention affects the pharmacokinetics and pharmacodynamics of conventional drugs. When microbial therapies (such as probiotics) interact with drugs, cross-toxicity may occur, leading to changes in drug efficacy, increased toxicity, or triggering of unexpected immune reactions. These interactions are complicated by the differences in the composition of microbial communities as affected by diet, environment, and species-specific factors, thereby influencing the results of microbial therapy and drug interaction [105]. Therefore, understanding these interactions is crucial for optimizing treatment strategies and improving the management outcomes of rheumatoid arthritis [110,111].

## 5. Translational Strategies from Human/Rodent Research to Veterinary Medicine

At present, there are still relatively few studies on RA in livestock and poultry animals. However, the existing findings have a positive promoting effect on veterinary research and practice.

The translational potential of human/rodent RA research to veterinary medicine is rooted in the evolutionary conservation of interactions between the gut microbiota and the immune system. Comparative microbiome analysis reveals shared dysbiosis patterns between human RA and animal joint lesions. As mentioned in Table 1 and Table 2, in human RA patients, we observed the enrichment of pro-inflammatory microbiota (such as *Escherichia*) and the reduction of SCFA-producing bacteria (such as *Faecalibacterium*), and similar changes were observed in chicks with inflammatory arthritis. This cross-species microbial signature suggests that therapeutic methods targeting the microbiota that are effective in humans may produce similar benefits in animals. For example, SCFAs produced by commensal bacteria such as *Bacteroides* and *Clostridium* exert anti-inflammatory effects in different species by promoting Treg cell differentiation and inhibiting Th17 cell-mediated inflammation. This mechanism has been verified in the rodent CIA model [112] and is considered promising for application in the intervention of inflammatory diseases in more species, such as cattle [113]. Similarly, the disruption of tight junction proteins in human RA patients is consistent with the observed digestive tract damage in rhesus monkeys with inflammatory arthritis [12], suggesting that there may be a conserved pathway between intestinal flora imbalance and systemic inflammation.

However, translational challenges arise from differences in immune responses and gut microbial ecology among species. There are significant differences in the immune systems and the degree of immune responses among different animals [114], so the dose and mode of intervention need to be adjusted. In addition, ruminants, such as cattle, carry unique gut microbiota, whose effects on fiber fermentation and SCFA production differ from those of humans [115]. This requires the development of ruminant-specific probiotics to optimize anti-inflammatory metabolite production in arthropathy-affected livestock.

To narrow the gap in translational research, future studies can focus on establishing standardized animal RA models to characterize the microbial-immune interactions in RA, and utilize cross-species proteomics and metabolomics to identify conserved biomarkers (such as SCFAs, Zonulin) related to the pathological mechanisms of RA, achieving the transformation of human diagnostic tools to veterinary medicine. At the same time, rodent models can be used for pre-screening the anti-RA effects of therapies targeting the microbiota (such as probiotics and FMT) and for further conducting clinical trials of veterinary-specific intervention measures to develop feasible measures for animal RA management, thereby meeting production needs and improving animal welfare.

## 6. Conclusions

In conclusion, the gut microbiota and the associated metabolites play an important role in the pathogenesis of RA. Modulating intestinal flora may provide a novel strategy for the prevention and treatment of RA. The information presented in this review will contribute to the elucidation of the pathogenesis of RA, enhance the efficacy of RA treatment in both humans and animals, and improve the life quality of patients and animals with RA to the extent possible.

## Figures and Tables

**Table 1:** The compositional and functional imbalance of the gut microbiota in arthritis animals.

Animals Included	Types of Arthritis	Classification Levels	Changes in Microbiota in Animals with Inflammatory Arthritis
Rhesus monkeys [12]	Pro-inflammatory arthritis (CHIKV infected)	Genus	*Prevotella* and *Sarcina* ↑ *Anaerostipes* and *Lactobacillus* ↓
		Species	*Prevotella copri* and *Bacteroides fragilis* ↑ *Butyricicoccus intestinisimiae* and *Streptococcus lutetiensis* ↓
Pigs [13]	Diet-induced exudative arthritis	Species	*Clostridium perfringens* ↑
Chicks [14]	Hypervirulent arthritis (*Salmonella Pullorum* infected)	Phylum	*Proteobacteria* ↑ *Firmicutes* ↓
		Genus	*Escherichia-Shigella* and *Klebsiell* ↑ *Lachnoclostridium* and *Blautia* ↓

↑ represents an increase in abundance, and ↓ represents a decrease in abundance.

**Table 2:** The compositional and functional imbalance of gut microbiota in RA patients.

Patients Included	Classification Levels	Changes in Microbiota in RA Patients
RA patients [4]	Genus	*Klebsiella* and *Escherichia* ↑ *Fusicatenibacter* and *Megamonas* ↓
Female patients with early RA [5]	Phylum	*Bacteroidetes* ↑ *Actinobacteria* ↓
	Genus	*Collinsella* ↓
Female RA patients [15]	Genus	*Bacteroides* and *Megamonas* ↑ *Prevotella* and *Gemmiger* ↓
RA patients [16]	Phylum	*Verrucomicrobia* and *Proteobacteria* ↑ *Bacteroidetes* ↓
	Genus	*Lactobacillus* and *Streptococcus* ↑ *Bacteroides* and *Faecalibacterium* ↓
RA patients [17]	Species	*Bifidobacterium longum* and *Dorea formicigenerans* ↑ *Faecalibacterium prausnitzii* and *Bacteroides* spp. ↓
RA patients [18]	Genus	*Eubacterium* and *Escherichia-Shigella* ↑
RA patients grouped into stages I–IV [19]	Species	*Escherichia coli* ↑ *Bacteroides uniformis* and *Bacteroides plebeius* ↓

↑ represents an increase in abundance, and ↓ represents a decrease in abundance.

**Table 3:** Effects of gut microbiota-derived metabolites on RA.

Metabolites of Intestinal Microorganisms	Associated Gut Microbes	Biological Functions
SCFAs [71,72]	*Clostridium*, *Bacteroides*, and *Eubacterium*	Decrease serum Zonulin concentration, increase the expression of tight junction protein, and restore the intestinal permeability
Tryptophan [73,74]	*Bifidobacterium*, *Clostridium*, *Escherichia coli,* and *Enterococcus*	Activate the AHR pathway, inhibit the activation and inflammatory response of intestinal immune cells, regulate the proliferation and differentiation of intestinal mucosal cells, and promote the repair and stability of mucosal barrier
Bile acid [75,76]	*Clostridium*, *Bacteroides,* and *Alistipes*	Promote the expression of tight junction proteins and blocking proteins and reduce the secretion of inflammatory cytokines (TNF-α and IL-6) to protect the intestinal barrier
Indole-3-aldehyde, Indole-3-acetic acid [77,78]	*Lactobacillus*, *Bifidobacterium,* and *Clostridium*	Affect the activity of Tregs cells, thereby affecting the balance of immune responses
Trimethylamine N-Oxide [79,80]	*Anaerocccus hydrongenalis*, *Clostridium asparagiforme,* and *Clostridium hathewayi*	Induce IL-1β, TNF-α, and IL-6, produce chemokines, and hinder bile acid synthesis and metabolism

**Table 4:** Summary of therapeutic interventions targeting gut microbiota in RA management.

Intervention Type	Specific Approach	Animal Model/Study Cohort	Observed Effects	Mechanism of Action
Probiotics	*Lactobacillus casei* [64]	Adjuvant-induced arthritis rats	Inhibited joint swelling, lowered arthritis scores, and prevented bone destruction	Increases *Lactobacillus* abundance and reduces pro-inflammatory cytokines
	*Lactiplantibacillus plantarum* [67]	CIA mice	Reduced autoantibody levels and alleviated joint damage	Reduces intestinal permeability and corrects microbial imbalance
	*Bacillus coagulans* [66]	Adult RA patients	Relieved pain, reduced total C-reactive protein, and improved patients’ self-assessment	Inhibits pathogenic bacteria and promotes SCFA production
FMT	Healthy donor fecal transfer [89]	A female RA patient	Decreased DAS28 score and dropped titer of RA	Restores the microbiota composition and inhibits Th17 cell activation
Dietary Interventions	Anti-inflammatory diet [104]	RA patients	Alleviated swelling and pain of the joint	Reduces pro-inflammatory cytokines
Pharmacological–Microbial Interactions	MTX [95]	RA patients	Altered *Bacteroides* and *Faecalibacterium* abundance and affected the metabolism of the microbial community	Modulates gut microbiota to reduce immune activation
	Traditional Chinese medicine (Jingfang Granules) [94]	RA rats	Restored intestinal tight junction proteins and mitigated tissue damage	Increases SCFA production and activates AMPK signaling

## Data Availability

Not applicable.

## Abbreviations

The following abbreviations are used in this manuscript:

CIA	Collagen-induced arthritis
DCs	Dendritic cells
FMT	Fecal microbiota transplantation
HIF	Hypoxia-inducible factor
ILC3	Type 3 innate lymphocytes
LPS	Lipopolysaccharide
MTX	Methotrexate
NETs	Neutrophil extracellular traps
NOX	NADPH oxidase
PAD	Peptidylarginine deiminase
RA	Rheumatoid arthritis
RANKL	Nuclear factor-κB ligand
SCFAs	Short-chain fatty acids
5-HIAA	5-hydroxyindole-3-acetic acid
5-HT	5-hydroxytryptamine
